# Huaier aqueous extract inhibits colorectal cancer stem cell growth partially via downregulation of the Wnt/β-catenin pathway

**DOI:** 10.3892/ol.2013.1145

**Published:** 2013-01-21

**Authors:** TAO ZHANG, KE WANG, JUNSHU ZHANG, XIAOLEI WANG, ZHIGANG CHEN, CHAO NI, FUMING QIU, JIAN HUANG

**Affiliations:** 1 Cancer Institute (Key Laboratory of Cancer Prevention and Intervention, National Ministry of Education, Provincial Key Laboratory of Molecular Biology in Medical Sciences), Second Affiliated Hospital, Zhejiang University School of Medicine, Hangzhou, Zhejiang 310009, P.R. China; 2 Departments of Oncology, Second Affiliated Hospital, Zhejiang University School of Medicine, Hangzhou, Zhejiang 310009, P.R. China; 3 Preventive and Health Care, Second Affiliated Hospital, Zhejiang University School of Medicine, Hangzhou, Zhejiang 310009, P.R. China

**Keywords:** Huaier aqueous extract, antitumor activity, colorectal cancer, cancer stem cells

## Abstract

The existence of cancer stem cells (CSCs) is central to the pathogenesis and therapy resistance of colorectal cancer. The aim of this study was to evaluate whether Huaier aqueous extract, a Chinese medicine, has efficacy against CSCs and to investigate the mechanisms of its anticancer effects. It was observed that the Huaier extract significantly inhibited the spheroid formation potential (P<0.05) and decreased the aldehyde dehydrogenase (ALDH)-positive cell population in colorectal primary cancer cells (P<0.05). Western blotting analysis and Wnt/β-catenin reporter assays revealed that the Huaier extract downregulated the Wnt/β-catenin self-renewal pathway. This is the first study to demonstrate that Huaier aqueous extract acts as an effective agent for eradicating colorectal CSCs and identifies the Wnt/β-catenin pathway as its potential target, which may be a new approach for colorectal cancer therapy.

## Introduction

Colorectal cancer (CRC) is a common lethal malignancy around the world. The initiation and progression of CRC is a complex process that results from the loss of the normal regulatory pathways between cell proliferation, differentiation and apoptosis. Previous studies have identified a small subset of cancer-initiating cells within tumors that drive tumor growth and recurrence, termed cancer stem cells (CSCs) (1–3). CSCs possess self-renewal capabilities and the ability to generate tumor bulk. Several signaling pathways appear to be key to the self-renewal behavior of CSCs, including the Wnt/β-catenin, Notch and Hedgehog pathways (4–6). The ultimate failure of numerous current cancer treatments, including chemo- and radiation therapy, is due to a failure to eliminate CSCs (7–11). The surviving CSCs regenerate recurrent tumors. Therefore, new drugs and novel therapies are required for the treatment of cancer patients. Several natural products have been demonstrated to be effective against CSCs, including curcumin, sulforaphane and epigallocatechin-3-gallate (12–14).

Huaier aqueous extract (obtained from *Trametes robiniophila*) has been used for the treatment of diseases such as viral hepatitis in China for many years (15). It is isolated from the extract of the officinal fungi and the effective ingredient has been identified as proteoglycan (containing 8.72% water, 12.93% amino acids and 41.53% polysaccharides) (16,17). It has been reported that Huaier extract has anticancer activity against various cancer types through the inhibition of tumor growth, induction of apoptosis and anti-angiogenic effects (16,18,19). There are, however, no studies dealing with the effect of Huaier extract on colorectal CSCs at present.

The Wnt/β-catenin pathway is one of the critical pathways demonstrated to mediate the self-renewal of CSCs. The activation of Wnt target genes depends on mediation by β-catenin, which enters the nucleus to transactivate the TCF/LEF transcription factor (20,21). The level of intracellular β-catenin is regulated by the axin-adenomatous polyposis coli-glycogen synthase kinase-3β complex and β-catenin is degraded via the ubiquitin-proteasome pathway (22,23). Activation of the Wnt/β-catenin pathway in CSCs has been shown to mediate the resistance to chemo- and radiation therapy (24,25). This indicates that the dysregulation of β-catenin is crucial in CSCs. If β-catenin transcriptional activity is markedly down-regulated, tumor growth is likely to be suppressed. Therefore, it is of great importance to find agents that are able to directly target this pathway and its downstream targets.

The present study examined the effects of Huaier aqueous extract on colorectal CSCs. The results showed that Huaier eliminated CSCs, partially by downregulating β-catenin and consequently inhibiting the Wnt pathway. The present study, for the first time, identified Huaier as an effective agent for eradicating CSCs and implicated the Wnt pathway as a potential target of Huaier in CRC.

## Materials and methods

### Materials

Huaier aqueous extract was obtained from Gaitianli Pharmacy Co. (Qidong, China). Dulbecco’s modified Eagle’s medium, nutrient mixture F-12 (DMEM/F12), was purchased from Invitrogen (Carlsbad, CA, USA). Fetal bovine serum (FBS) was supplied by Sijiqing Biological Engineering Materials Co., Ltd. (Hangzhou, China). The anti-β-catenin (1:3,000), anti-cyclin D1 (1:3,000) and anti-β-actin (1:1,000) antibodies were purchased from Cell Signaling Technology (Beverly, MA, USA). The collagenase and hyaluronidase were obtained from Sigma Chemical (Balcatta, WA, Australia).

### Tumor cell preparation

Primary CRC cells (T1 and T2 cells) were established from patients’ cancer tissues following surgery as described previously (26). In brief, resected CRC tissues were obtained in accordance with the Research Ethics Board on Human Experimentation at the Second Affiliated Hospital, Zhejiang University School of Medicine (Hangzhou, China) from two patients with informed consent. The histological diagnosis was based on microscopic features of the carcinoma cells. The cancer tissues were intensively washed four times in PBS solution containing antibiotics. Enzymatic digestion was performed using collagenase (1.5 mg/ml) and hyaluronidase (20 mg/ml) in PBS for 1 h. The cancer cells were then used for culturing in DMEM/F12 supplemented with 10% FBS and 1X antibiotic-antimycotic. The cells were finally incubated at 37°C in a 5% CO_2_ humidified incubator. Cultures contaminated with fibroblasts were removed and cancer cells were identified in NOD/SCID mice.

### Viability assays

The proliferation rates and sensitivity to Huaier extract were assessed by MTS assays using the CellTiter 96 Aqueous MTS kit (Promega, Fitchburg, WI, USA). The colorectal primary cancer cells were seeded in 100 *μ*l medium at a density of 2-5×10^4^ cells per well in 96-well plates (Corning, New York, NY, USA). Following exposure to the Huaier extract for 48 h, the MTS assay was performed according to the manufacturer’s instructions.

### Detection of Huaier-induced changes in cell morphology

CRC cells were plated in 24-chamber culture plates at 10,000 cells per well, allowed to adhere and incubated with the Huaier aqueous extract at 8 mg/ml for 48 h. The morphology of the cells was visualized and photomicrographs were obtained with an Olympus light microscope (×10).

### Spheroid formation assay

Cells were cultured in DMEM/F12 basal serum-free medium supplemented with 20 ng/ml EGF (Invitrogen), 10 ng/ml bFGF, B27, 100 U/ml penicillin and 100 *μ*g/ml streptomycin. Single cells were prepared by enzymatic dissociation and seeded at low densities (100–200 cells/well) in 96-well low-adhesion plates (Corning). Various concentrations of the Huaier extract were added. After 14 days of culturing, the number and size of spheroids were assessed.

### Aldehyde dehydrogenase (ALDH) assay

Colorectal CSCs are highly enriched with cells having a high ALDH enzyme activity (27). The Aldefluor assay was performed using an Aldefluor kit (Stemcell Technologies, Durham, NC, USA). Single cells obtained from cell cultures were incubated in an Aldefluor assay buffer containing an ALDH substrate for 40 min at 37°C according to the manufacturer’s instructions. A fraction of the cells was incubated with the ALDH inhibitor diethylaminobenzaldehyde under the same conditions as a negative control. The cells were analyzed with a FACSCalibur instrument (Becton Dickinson, Franklin Lakes, NJ, USA).

### Protein isolation and western blot analysis

The cells were treated with Huaier aqueous extract (2 or 4 mg/ml) for 48 h. At the end of the incubation period, the cells were harvested and lysed using the radioimmunoprecipitation assay buffer [20 mmol/l Tris-HCl, 150 mmol/l NaCl, 1% NP-40, 5 mmol/l EDTA and 1 mmol/l Na_3_VO_4_ (pH 7.5)] supplemented with a protease inhibitor cocktail. Proteins were detected by western blot analysis using anti-β-catenin, anti-cyclin D1 and anti-β-actin. Goat anti-rabbit IgG conjugated to horseradish peroxidase (HRP) was used as the secondary antibody. Immunoreactive bands were detected using a chemiluminescent substrate.

### TCF/LEF transcription assay

To evaluate the TCF/LEF transcriptional activity induced by activated β-catenin, TOPflash and the negative control FOPflash, a pair of luciferase reporter constructs, were used. The reporter system was a gift from Dr Yongliang Zhu (Zhejiang University School of Medicine). In brief, the cells were transiently infected with either TOPflash reporter plasmids (10 *μ*g/100 *μ*l) or FOPflash plasmids (10 *μ*g/100 *μ*l) together with Renilla-tk plasmids (1 *μ*g/100 *μ*l; encoding Renilla luciferase). After 24 h, various concentrations of the Huaier extract were added and the cells were continually incubated in the same medium for 24 h. Cells were washed with PBS and the luciferase activity was measured with the dual-luciferase reporter assay system (Promega).

### Statistical analysis

The statistical analysis was performed with GraphPad Prism 5.0 software and statistical differences were determined by one-way ANOVA. The data were expressed as the mean ± standard deviation and all experiments were performed in triplicate. P<0.05 was considered to indicate a statistically significant difference.

## Results

### Huaier aqueous extract suppresses the growth of CRC cells

**Figure 1:**
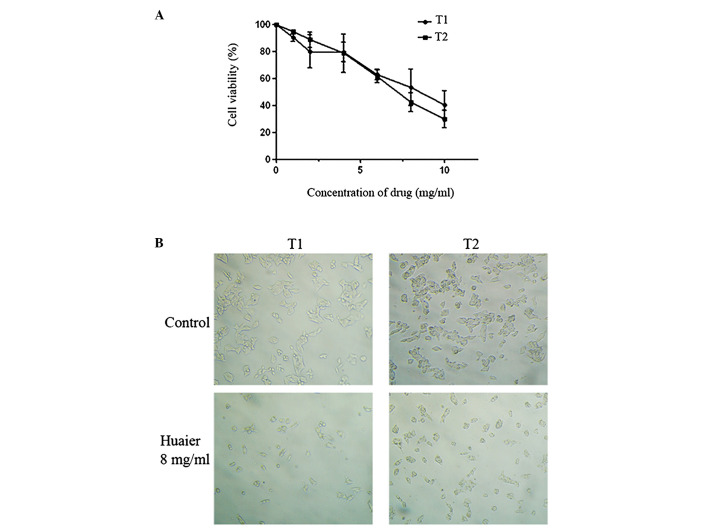
Huaier aqueous extract suppresses cell viability in colorectal cancer cells. (A) Dose-response curves of primary colorectal cancer cells (T1 and T2 cells) treated with Huaier extract for 48 h. The antiproliferative effect of the Huaier aqueous extract was measured using the MTS assay. Bars denote the standard deviation. (B) Huaier extract caused morphological changes in the colorectal cancer cells. The images of colorectal cancer cells were obtained 48 h after the addition of Huaier extract.

To evaluate the biological activity of Huaier aqueous extract on cancer cells, cell viability was measured using the MTS assay following treatment with various concentrations of the extract. Cell survival decreased with increasing concentrations of the Huaier extract and the IC_50_ was determined to be ∼8 mg/ml for T1 and T2 cells (colorectal primary cells; Fig. 1A). The results showed the Huaier extract to be an antiproliferative agent against CRC cells.

In addition to the antiproliferative effects, the morphological effects of the Huaier extract on CRC cells were also investigated. The control cells were epithelial cells with radial spread and large cell size, indicating typical epithelial morphology (Fig. 1B). Following the addition of the Huaier extract, the cell morphology was markedly altered. In the majority of the Huaier-treated colorectal cells, the membranes shrank and the cells became irregularly shaped or round with no visible radial spread. These alterations were accompanied by a decrease in cell number.

### Huaier aqueous extract inhibits the formation of spheroids

**Figure 2:**
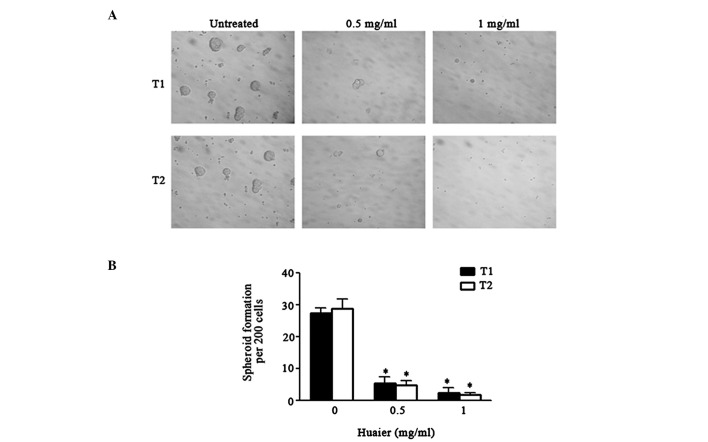
Huaier aqueous extract inhibits the formation of spheroids. (A) Colorectal cancer cells (T1 and T2 cells) were incubated with the Huaier extract for 14 days under spheroid-forming conditions. The size of the spheroids was then evaluated under a microscope. (B) Quantification of spheroid-formation with colorectal cancer cells treated as in (A). Bars denote the standard deviation. ^*^P<0.05 vs. untreated cells.

To evaluate its direct effects on the formation of spheroids, spheroidal growing cells were treated with Huaier aqueous extract. CRC cells were exposed to varying concentrations of the Huaier extract and then cultured for 14 days. The Huaier extract inhibited spheroid formation by the cancer cells (Fig. 2). Not only did the number of spheroids decline significantly, but also the size of the spheroids was reduced (P<0.05). It was noteworthy that the concentrations of Huaier capable of suppressing spheroid formation were ∼15-fold lower than those exhibiting antiproliferative effects in the MTS assay (IC_50_, ∼8 mg/ml for T1 and T2 cells).

### Huaier aqueous extract effectively eliminates ALDH-positive cells in vitro

**Figure 3:**
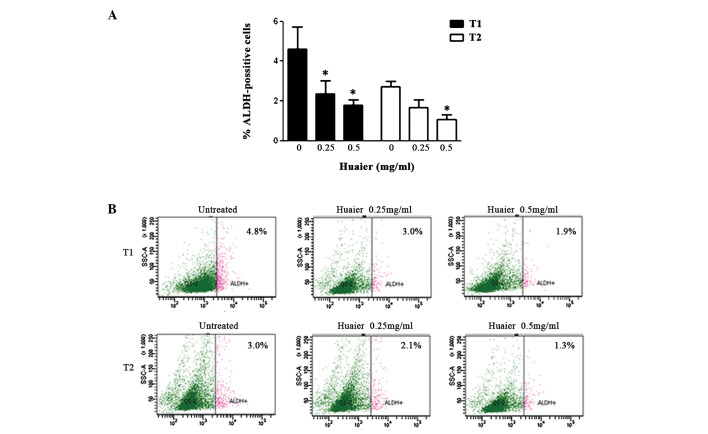
Huaier aqueous extract decreases the ALDH-positive cell population. Colorectal cancer cells (T1 and T2 cells) were treated with Huaier extract for seven days and subjected to the Aldefluor assay. (A) Proportion of the ALDH-positive cell subpopulation in total cancer cells. Bars denote the standard deviation.^*^P<0.05 vs. untreated cells. (B) Representative flow cytometry. ALDH1, aldehyde dehydrogenase.

ALDH1 activity, a marker associated with CSCs, was further tested by the Aldefluor assay. Cell populations with high ALDH activity have been demonstrated to enrich CSCs. Long-term treatment with Huaier extract for seven days led to a selective decrease in the ALDH-positive populations of the CRCs (Fig. 3; P<0.05). This finding demonstrated that the Huaier extract was able to eliminate CSCs *in vitro*. A notable observation was that the Huaier extract was able to kill CSCs at concentrations (0.25–0.5 mg/ml) that hardly affected the bulk cancer cells, suggesting that it may preferentially target CSCs compared with the bulk cancer cells.

### Huaier aqueous extract downregulates the Wnt/β-catenin pathway

**Figure 4:**
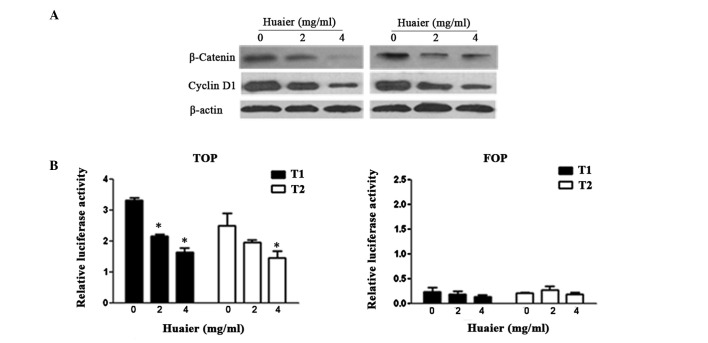
Huaier aqueous extract downregulates the Wnt/β-catenin pathway. (A) Expression of β-catenin and cyclin D1 was detected by western blot analysis in colorectal cancer cells either untreated or treated with Huaier extract for 48 h. (B) TOP/FOPflash reporter transiently infected colorectal cancer cells were treated with Huaier extract for 24 h. The Huaier extract decreased the percentage of TOP reporter. Bars denote the standard deviation (^*^P<0.05).

The Wnt/β-catenin pathway is a key pathway in CSC self-renewal that is activated via β-catenin and results in the phosphorylation of numerous downstream molecules. Whether the Huaier extract altered the Wnt/β-catenin pathway in human CRC cells was studied. The β-catenin protein was constitutively expressed in cancer cells. Treating the CRC cells with Huaier led to dose-dependent downregulation of the levels of total β-catenin protein and also decreased the expression of cyclin D1 (Fig. 4A), one of the Wnt/β-catenin target genes. To further investigate whether the downregulation of β-catenin reduced the activity of downstream molecules, a TOP/FOP flash reporter system was used. Activation of TCF/LEF in the nucleus mediated by β-catenin was detected and quantified with a luciferase assay system. A decrease in the TOP levels was observed in the CRC cells treated with Huaier extract (Fig. 4B; P<0.05). These data indicate that the downregulation of the Wnt/β-catenin self-renewal pathway may be a potential target of the Huaier extract.

## Discussion

The anticancer effects of Huaier aqueous extract, a traditional Chinese medicine obtained from the extract of the officinal fungi, has been evaluated in various types of cancer. For instance, treatment with Huaier has been shown to inhibit the proliferation of breast cancer cells by inducing apoptosis (16). Additionally, it has been suggested that Huaier has various biological activities related to metastasis inhibition, immune system activation and drug resistance reversal (15). These findings provide a clear rationale for investigating the preventive and therapeutic properties of Huaier in clinical trials. Huaier has been used for treating primary liver cancer for many years in China (28). However, the effects of Huaier on colorectal CSCs and its mechanism require further validation.

In the present study, the Huaier extract demonstrated potent anti-CSC effects in CRC cells. At present, although there are no exact criteria that directly identify CSCs, several techniques are used to isolate and characterize colorectal CSCs. One method is to use a particular stem cell marker such as ALDH1 (29,30). Unlike the CD molecules, ALDH1 is a recently identified cancer stem cell marker which has greater specificity than CD133 and CD44 for colorectal CSCs (31,32). Previous studies have shown that in normal crypts, ALDH1 positive cells are sparse and limited to the normal crypt bottom. During the conversion from normal epithelium to adenoma, the number of ALDH-positive cells increases and distributes in the crypt and as few as 25 cells are able to form a tumor in NOD/SCID mice. Thus, the Aldefluor assay was used to evaluate the effects of the Huaier extract in eliminating colorectal CSCs. The present results revealed that the Huaier extract was able to kill the tumor-initiating ALDH-positive cells. Another method of characterizing CSCs is spheroid culture under the absence of serum and without attachment to culture plates. CSCs form spheroids while differentiated cells fail to survive (33,34). The Huaier extract was observed to inhibit the spheroid-forming capacity of CRC cells. These results suggest that Huaier is able to eliminate the small percentage of cells in a tumor responsible for chemo-resistance and recurrence.

In the current study, it was demonstrated that Huaier was able to target and inhibit the Wnt/β-catenin pathway with a concomitant decrease in β-catenin expression, thereby manifesting its anticancer mechanism and effects. Of particular note, the total β-catenin and cyclin D1 levels were downregulated in all cells studied at 48 h. These results suggested that the Wnt pathway may be considered as a potential target of Huaier for preventive and therapeutic interventions in CRC. The Wnt/β-catenin pathway is closely associated with self-renewal and chemoresistance in CRC. Previous evidence has further demonstrated the role of the overactive Wnt/β-catenin pathway in colorectal carcinogenesis (35). In the present study, the results showed downregulation of the Wnt pathway, along with a decrease in the levels of β-catenin and cyclin D1, making Huaier an attractive potential drug for cancer therapy.

The antitumor activity of Huaier aqueous extract has been reported *in vivo*(36); at 2.5 g/kg per day it was able to significantly suppress tumor growth and showed no cytotoxicity in the treated mice, suggesting that Huaier is effective and safe. In addition, commercial Huaier products, (also called Huaier particles; code number approved by SFDA: Z20000109) have also been used clinically, with effects on various types of human cancer, including lung, esophageal, gastric and liver carcinomas. It is thus possible that Huaier may produce desirable antitumor effects in CRC. However, further studies of Huaier and its potential clinical applications are necessary and important.

The present study demonstrated that Huaier aqueous extract is able to target colorectal CSCs and inhibit the spheroid formation potential and ALDH-positive cell population. One of the mechanisms for the therapeutic effects of Huaier may be the downregulation of the Wnt/β-catenin self-renewal pathway. The present study suggests that the use of Huaier may be a good choice for treating CRC.
